# Fibroblast growth factor 21 Ameliorates diabetes-induced endothelial dysfunction in mouse aorta via activation of the CaMKK2/AMPKα signaling pathway

**DOI:** 10.1038/s41419-019-1893-6

**Published:** 2019-09-11

**Authors:** Lei Ying, Na Li, Zhengyue He, Xueqin Zeng, Yan Nan, Jiantong Chen, Peipei Miao, Yunyun Ying, Wei Lin, Xinyu Zhao, Lu Lu, Mengke Chen, Wei Cen, Tonglin Guo, Xiaokun Li, Zhifeng Huang, Yang Wang

**Affiliations:** 1 Department of Pathophysiology, School of Basic Medical Sciences, Wenzhou Medical University, 325035 Wenzhou, Zhejiang China; 2 Department of Pathology, Wenzhou Central Hospital, 325035 Wenzhou, Zhejiang China; 3 Department of Pathology, Suining Central Hospital, 629000 Suining, Sichuan China; 4 The Second Affiliated Hospital and Yuying Children’s Hospital, Wenzhou Medical University, 325035 Wenzhou, Zhejiang China; 5 Department of Pharmacy, the Second People’s Hospital of Pingyang, 325035 Wenzhou, Zhejiang China; 6 The First Affiliated Hospital & School of the First Clinical Medical Sciences, Wenzhou Medical University, 325035 Wenzhou, Zhejiang China; 7 School of Pharmaceutical Sciences, Wenzhou Medical University, 325035 Wenzhou, Zhejiang China; 8 School of Ophthalmology and Optometry, Wenzhou Medical University, 325035 Wenzhou, Zhejiang China

**Keywords:** Aortic diseases, Diabetes complications

## Abstract

Endothelial dysfunction initiates and exacerbates hypertension, atherosclerosis and other cardiovascular complications in diabetic mellitus. FGF21 is a hormone that mediates a number of beneficial effects relevant to metabolic disorders and their associated complications. Nevertheless, it remains unclear as to whether FGF21 ameliorates endothelial dysfunction. Therefore, we investigated the effect of FGF21 on endothelial function in both type 1 and type 2 diabetes. We found that FGF21 reduced hyperglycemia and ameliorated insulin resistance in type 2 diabetic mice, an effect that was totally lost in type 1 diabetic mice. However, FGF21 activated AMPKα, suppressing oxidative stress and enhancing endothelium-dependent vasorelaxation of aorta in both types, suggesting a mechanism that is independent of its glucose-lowering and insulin-sensitizing effects. In vitro, we identified a direct action of FGF21 on endothelial cells of the aorta, in which it bounds to FGF receptors to alleviate impaired endothelial function challenged with high glucose. Furthermore, the CaMKK2-AMPKα signaling pathway was activated to suppress oxidative stress. Apart from its anti-oxidative capacity, FGF21 activated eNOS to dilate the aorta via CaMKK2/AMPKα activation. Our data suggest expanded potential uses of FGF21 for the treatment of vascular diseases in diabetes.

## Introduction

The incidence of diabetes mellitus is increasing at an alarming rate, imposing tremendous suffering and economic burdens on patients. The major hazards of diabetes are its complications, especially cardiovascular diseases that are primary causes of mortality^1^. Among these vasculopathies, endothelial dysfunction is thought to be the early event initiated by metabolic stresses, including insulin resistance, hyperglycemia and dysregulated lipid metabolism^2–4^. Endothelial cells in vasculature play essential roles in the prevention of inflammation, regulation of vascular tone, and maintenance of the balance between coagulation and anticoagulation^5^. Conversely, the impairment of endothelium in blood vessels leads to hypertension, atherosclerosis and other cardiovascular diseases^2,3,5^. Therefore, the control of endothelial function during diabetes is an efficient way to prevent severe cardiovascular damage.

Oxidative stress occurring in metabolic disease is a major risk factor for endothelial dysfunction^2,3,5^. Many pathological changes induce oxidation in endothelial cells, thereby increasing production of reactive oxygen species (ROS)^2,3,5^. Then, accumulating amounts of ROS further lead to impairment of mitochondrial function and exacerbate the oxidative burden for the cell^2,3,5^. Simultaneously with decreased levels of antioxidant enzymes, the oxidative stress becomes more severe and endothelial function is largely inhibited due to insufficiency of nitric oxide (NO) bioavailability by forming peroxynitrite, inducing endothelial nitric oxide synthase (eNOS) uncoupling and increasing lipid peroxidation products^5,6^. Therefore, therapeutic approaches targeting oxidative stress are efficient methods to ameliorate impairments in vascular endothelial cells due to diabetic challenge.

Identified as the 21st member of FGF superfamily in 2000, the endocrine hormone fibroblast growth factor 21 (FGF21) has attracted considerable attention regarding the management of metabolic diseases as well as their complications^7–9^. FGF21 has been proposed to be a sensitive stress signal for maintaining metabolic homeostasis in both physiological and pathological conditions^8^. In most physiological stress responses, FGF21 functions as a hepatokine acting on adipose tissues to regulate energy homeostasis^10–12^. FGF21 also acts in fat tissue to maintain body temperature, in an autocrine or paracrine manner^13,14^. In metabolic disorders such as obesity and type 2 diabetes (T2D), high levels of FGF21 are thought to represent compensatory responses to maintain metabolic homeostasis, which also represents its resistant state^15,16^. In addition to its function as a stress response hormone, the metabolic benefits of the pharmacological use of FGF21 have been well studied in rodents, nonhuman primates and humans, where it dramatically alleviated obesity and fatty liver disease^8,9^. Recently, several lines of evidence have shown that the vascular system might be a target for FGF21. For example, FGF21 protects against atherosclerosis by inducing adiponectin to inhibit neointima formation and macrophage inflammation in blood vessels and suppresses hepatic cholesterol synthesis to attenuate hypercholesterolemia^17^. FGF21 also acts adipocytes and renal cells to promote metabolism of angiotensin II and to mitigate hypertension and vessel injury^18^. FGF21 is also a potent anti-oxidative agent, protecting heart and kidney in various conditions^19,20^. We therefore hypothesized that FGF21 might exert anti-oxidative activities in vascular endothelia cells, and that these effects could be exploited to treat reduced NO bioavailability and associated cardiovascular diseases.

To test this hypothesis, we generated recombinant human FGF21 (rFGF21) and investigated its effects on endothelial function both in vivo and in vitro. These experiments identified FGF21 as an efficient anti-oxidative protein acting on vascular endothelial cells that reduced oxidative stress, enhanced eNOS activity and improved vessel relaxation, probably via CaMKK2/AMPKα activation. Our data have important implications for FGF21 as a therapeutic approach for alleviation of endothelial dysfunction in diabetes.

## Results

### FGF21 improves endothelial dysfunction of aorta in Type 2 diabetic mice

**Fig. 1:**
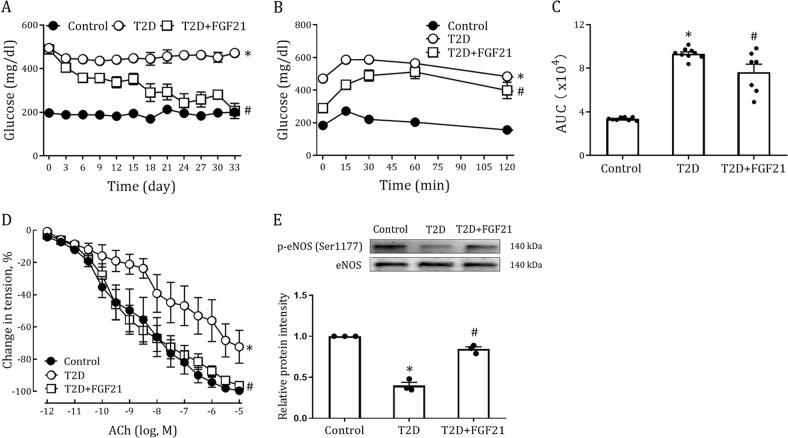
Long-term treatment of HFD-STZ-Induced T2D mice with rFGF21 improves hyperglycemia, insulin resistance and endothelium-dependent relaxation of aorta. **a–e** HFD-STZ-induced T2D mice were treated for 33 days with rFGF21 (0.5 mg/kg body weight) or buffer control. **a** Random fed blood glucose (*n* = 10). **b** IPGTT over the course of 120 mins (*n* = 7–9). **c** Integrated AUC for changes in blood glucose levels (*n* = 7–9). **d** Dose-dependent relaxation of aorta to ACh (*n* = 5–7). **e** Phosphorylation level of eNOS in aortas as determined by western blot analysis (upper panel) and quantitation using ImageJ software (lower panel) (*n* = 3). All data are presented as mean ± SEM. *p < 0.05 vs Control; ^#^*p* < 0.05 vs T2D

Endothelial dysfunction in conduit arteries induced by metabolic disorders is a well-established antecedent of hypertension and atherosclerosis^2–4^. Although the therapeutic effects of FGF21 on the latter two vasculopathies have been well studied previously^17,18^, it remains unclear whether FGF21 ameliorates impaired endothelial function in metabolic diseases such as T2D. Therefore, we established a high-fat diet (HFD)-streptozotocin (STZ) induced T2D mouse model and injected these mice intraperitoneally (i.p.) on 33 consecutive days with rFGF21 (0.5 mg/Kg body weight). As previously reported^21^, we found that rFGF21 (thought to be an insulin sensitizer) induced a substantial glucose-lowering effect in T2D mice that was associated with improved insulin resistance (Fig. 1a–c). Dysregulated lipid metabolism in these mice was also restored by chronic rFGF21 treatment (as revealed by suppressed serum levels of low-density lipoprotein (LDL), cholesterol, triglyceride and free fatty acid (FFA) and upregulated level of high-density lipoprotein (HDL)) (Fig. S1A–E), in addition to reduced body weights (Figure S2A).

Because hyperglycemia, insulin resistance and hyperlipidemia are major risk factors for vascular endothelial dysfunction, we next examined whether FGF21 rescued impaired endothelial function in T2D^2,3^. As shown by dose-dependent relaxation to acetylcholine (ACh), the impaired endothelium-dependent dilation of aorta in T2D mice was largely improved by chronic rFGF21 treatment (Fig. 1d), in accordance with increased phosphorylation levels and activation of eNOS (Fig. 1e).

**Fig. 2:**
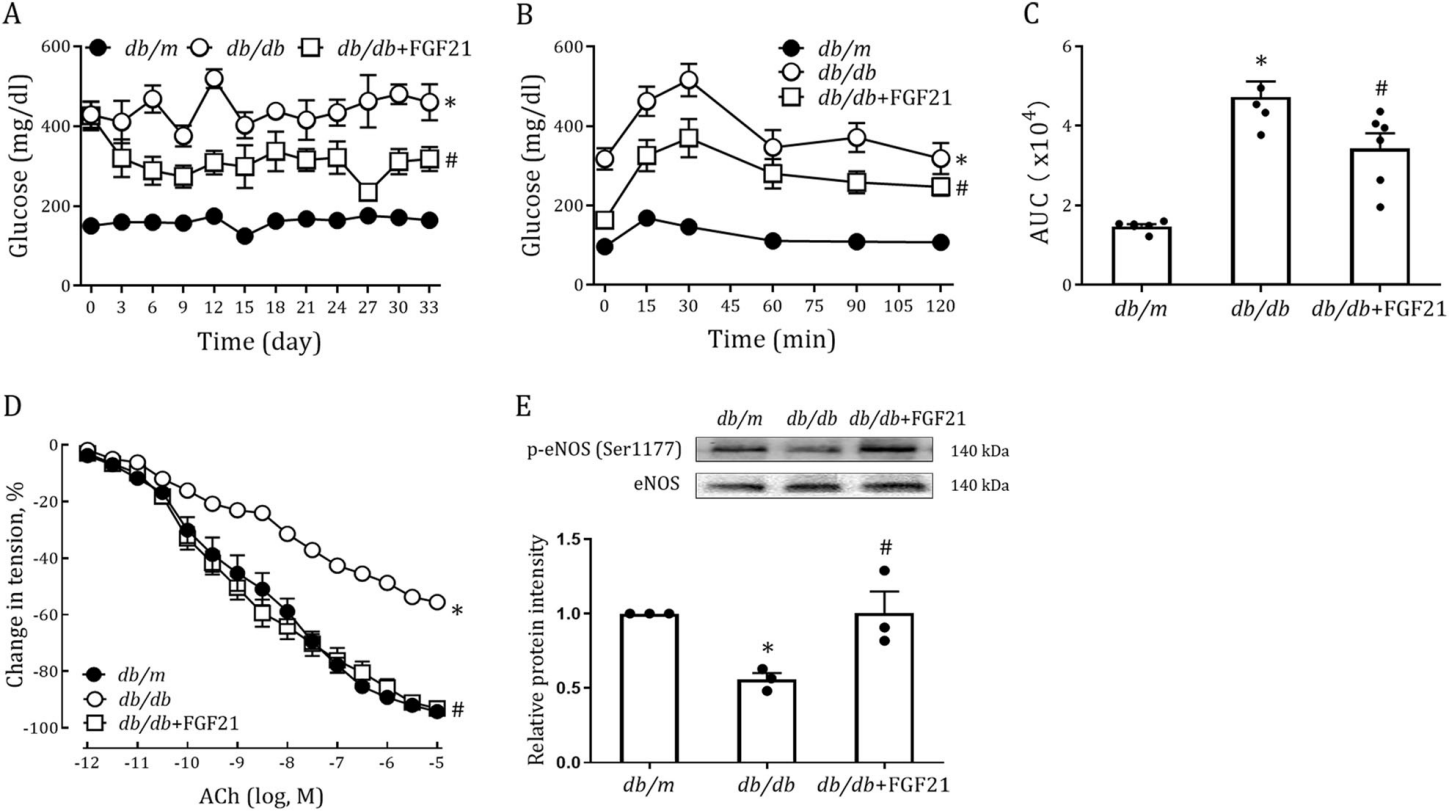
Long-term treatment of *db/db* mice with rFGF21 improves hyperglycemia, insulin resistance and endothelium-dependent relaxation of aorta. **a–e** *db/db* mice were treated for 33 days with rFGF21 (0.5 mg/kg body weight) or buffer control; littermate *db/m* mice served as controls. **a** Random fed blood glucose (*n* = 5–6). **b** IPGTT over the course of 120 mins (*n* = 5–6). **c** Integrated AUC for changes in blood glucose levels (*n* = 5–6). **d** Dose-dependent relaxation of aorta to ACh (*n* = 5–6). **e** Phosphorylation level of eNOS in aortas as determined by western blot analysis (upper panel) and quantitation using ImageJ software (lower panel) (*n* = 3). All data are presented as mean ± SEM. *p < 0.05 vs Control; ^#^*p* < 0.05 vs *db/db*

These beneficial effects of FGF21 were further investigated in another genetic induced (*db/db*) T2D mouse model which also received 33 consecutive days of i.p. injection with rFGF21 (0.5 mg/Kg body weight). Consistent with this result, we also found that hyperglycemia, insulin resistance and endothelium dysfunction in *db/db* mice were markedly improved by rFGF21 treatment in the same way (Fig. 2a–e), whereas there was little change in body weight (Fig. S2B).

### FGF21 alleviates endothelial dysfunction in aorta of type 1 diabetic mice

**Fig. 3:**
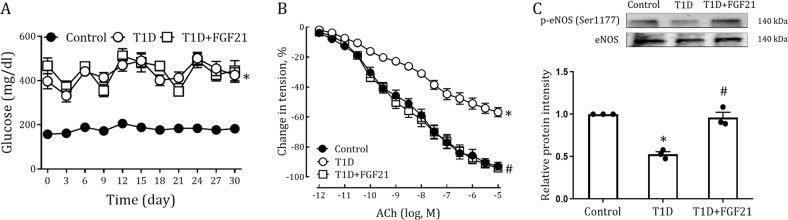
Long-term treatment of T1D mice with rFGF21 improves endothelium-dependent relaxation of aorta. **a–c** STZ-induced T1D mice were treated for 30 days with rFGF21 (0.5 mg/kg body weight) or buffer control; littermate *db/m* mice served as controls. **a** Random fed blood glucose (*n* = 8–9). **b** Dose-dependent relaxation of aorta to ACh (*n* = 7–8). **c** Phosphorylation level of eNOS in aortas as determined by western blot analysis (upper panel) and quantitation using ImageJ software (lower panel) (*n* = 3). All data are presented as mean ± SEM. *p < 0.05 vs Control; ^#^*p* < 0.05 vs T1D

Although its pathogenesis is quite different from that of T2D, type 1 diabetes (T1D) is also a major risk factor for impaired endothelial function mediated by hyperglycemia^3,22^. To explore whether the therapeutic effects of FGF21 on vascular function applied to T1D, we established an STZ-induced T1D mouse model and treated these mice with 30 days of rFGF21 injection (i.p., 0.5 mg/Kg body weight). Unlike in T2D mice, FGF21 failed to reduce high blood glucose in T1D mice with stable body weights (Fig. 3a, S2C). Nevertheless, it is interesting that FGF21 effectively alleviated T1D-induced aorta dysfunction as reveled by improved vascular relaxation and enhanced eNOS activity (Fig. 3b, c). These data suggest that FGF21 ameliorated endothelial dysfunction in T1D by mechanisms independent of its glucose-lowering and insulin-sensitizing effects.

### The protective effects of FGF21 on diabetic endothelial dysfunction involve antioxidative mechanisms

**Fig. 4:**
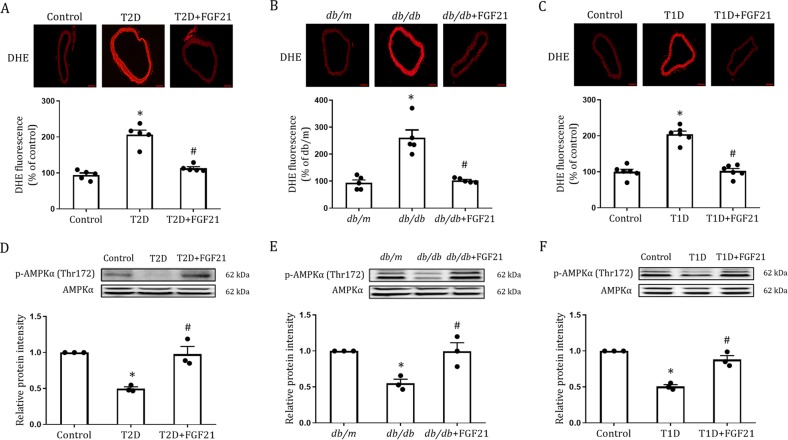
Long-term rFGF21 treatment alleviates oxidative stress and enhances AMPK signaling in aortas of HFD-STZ-induced T2D, *db/db* and T1D mice. **a–c** Immunofluorescent DHE staining of aortas from HFD-STZ-induced T2D. **a** (33 days) (*n* = 5), *db/db* (**b**) (33 days) (*n* = 5) or T1D mice (**c**) (30 days) (*n* = 6) chronically treated with rFGF21 (0.5 mg/kg body weight). The upper panel shows DHE staining and the lower panel shows quantitation using ImageJ software. Scale bars, 100 μm. **d–f** Phosphorylation levels of AMPKα in aortas from T2D (A) (33 days), *db/db* (B) (33 days) or T1D mice (C) (30 days) chronically treated with rFGF21 (0.5 mg/kg body weight) as determined by western blot analysis (upper panel) and quantitation using ImageJ software (lower panel) (*n* = 3). All data are presented as mean ± SEM. *p < 0.05 vs Control; ^#^*p* < 0.05 vs T2D, *db/db* or T1D

Oxidative stress initiated and exacerbated by metabolic disorders in both T1D and T2D is thought to be a primary cause of endothelial dysfunction^2,3,5^. Therefore, we next analyzed the oxidative status of aortas in rFGF21-treated HFD-STZ-induced T2D, db/db and T1D mice by immunostaining with dihydroethidium (DHE). Immunofluorescence analyses showed significantly fewer DHE-positive cells in all three mouse models treated with rFGF21 than in vehicle-treated mice (Fig. 4a–c).

A growing body of evidence has shown that adenosine 5′-monophosphate (AMP)-activated protein kinase (AMPK) plays a key role in maintaining oxidative homeostasis in endothelial cells of conduit arteries challenged with metabolic stress^23,24^. We measured phosphorylation levels of AMPKα in aortas from all diabetic mouse models and found that rFGF21 substantially rescued impaired AMPKα activity in these mice (Fig. 4d–f). Taken together, these data suggest that FGF21 may ameliorate endothelial dysfunction in diabetic mice via AMPKα-mediated inhibition of local oxidative stress in mouse aorta.

### FGF21 Ameliorates endothelial dysfunction by inhibiting oxidative stress via CaMKK2/AMPKα activation

**Fig. 5:**
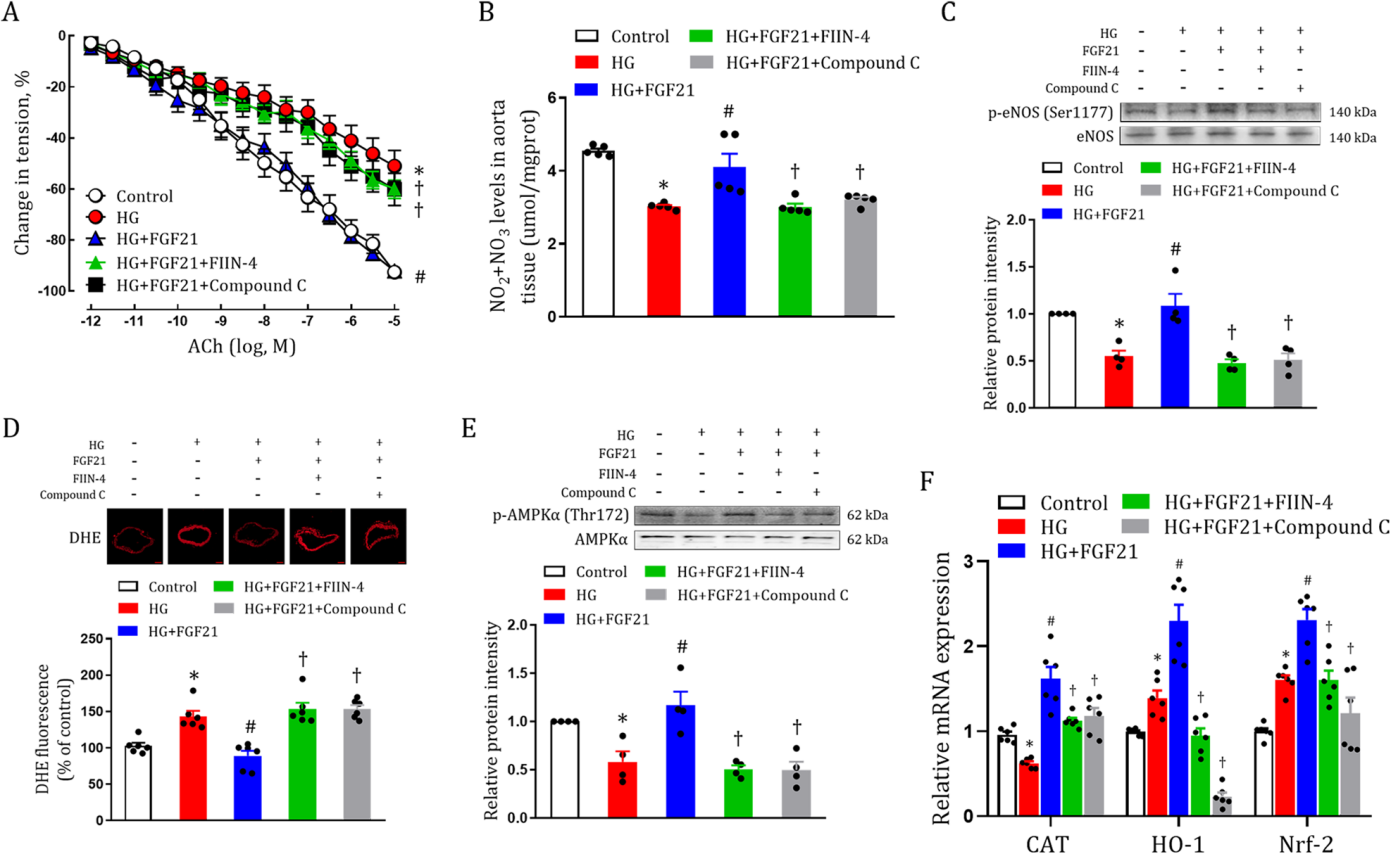
RFGF21 improves endothelium-dependent relaxation, alleviates oxidative stress and enhances AMPK signaling in aortas challenged with HG. **a–e** Aortas isolated form C57BL/6 J mice in Kreb’s buffer were pretreated with FIIN-4 (10 μM) or Compound C (10 μM) for 30 mins and exposed to either HG (30 mM) alone or HG plus rFGF21 (0.01 mg/ml) for an additional 2 h. **a** Dose-dependent relaxation to ACh (*n* = 8–18). **b** NO_2_ and NO_3_ levels stimulated by ACh (6 × 10^−8^ M) for 3 mins (*n* = 5). **c** Phosphorylation level of eNOS stimulated by ACh (6 × 10^−8^ M) for 3 mins as determined by western blot analysis (upper panel) and quantitation using ImageJ software (lower panel) (*n* = 4). **d** Immunofluorescent DHE staining (*n* = 6). The upper panel shows DHE staining and the lower panel shows quantitation using ImageJ software. Scale bars, 100 μm. **e** Phosphorylation level of AMPKα as determined by western blot analysis (upper panel) and quantitation using ImageJ software (lower panel) (*n* = 4). **f** mRNA levels of CAT, HO-1 and Nrf-2 as determined by real-time PCR analysis (*n* = 6). All data are presented as mean ± SEM. *p < 0.05 vs Control; ^#^*p* < 0.05 vs HG; ^†^*p* < 0.05 vs HG + FGF21

The animal studies suggested that there are some mechanisms involved in FGF21-mediated alleviation of endothelial dysfunction that are independent of reducing hyperglycemia and improving insulin resistance. Because endothelial cells express fibroblast growth factor receptor 1 (FGFR1) and β-klotho (primary receptors and co-receptors mediating the biological functions of FGF21) (Fig. S3A, B)^25–27^, one possibility is that FGF21 may directly bind with the corresponding receptor to mediate its therapeutic effects. Therefore, we established an in vitro model in which aorta was isolated from normal mice and challenged with high glucose (HG) alone or HG plus rFGF21. In this model, the high glucose condition was maintained throughout rFGF21 treatment that was devoid of exogenous insulin, partially mimicking effects in T1D mice. We found that endothelium-dependent relaxation was severely impaired by 2 h of HG incubation, and was reversed by co-administration with rFGF21 (Figs. 5a, S4A). Consistently, reduced NO oxide release, dampened eNOS activity and enhanced oxidative stress by HG were all ameliorated by rFGF21 (Figs. 5b–d, S4B-D), in parallel with the activation of AMPKα (Figs. 5e, S4E). These results were further reinforced by using a potent FGF receptor antagonist (FIIN-4)^28^, that blocked almost all the beneficial effects on endothelial function associated with improved eNOs activity, increased NO release and correspondingly enhanced relaxation of the aorta and reduced oxidative stress (Fig. 5a–e).

This *in vitro* model further strengthened the notion that AMPKα plays an important role in FGF21-mediated improvement in endothelial function. Using an AMPK-selective inhibitor (Compound C)^29^, we found that restoration of aorta relaxation (associated with enhanced eNOs activity and NO release) and redox homeostasis (associated with reduced ROS) by rFGF21 in HG situation were potently abrogated (Fig. 5a–e). To validate the role of AMPK in FGF21-mediated alleviation of endothelial dysfunction induced by HG, we used AMPKα siRNA to knockdown its expressions in human umbilical vascular endothelial cells (HUVECs). Consistently, we found that activations of AMPKα, Acetyl-CoA carboxylase (ACC) (a downstream target of AMPK)^30^ and eNOS by rFGF21 in HUVECs were markedly compromised by reduced AMPK expression (Fig. S5A–D). We further explored the downstream antioxidant signals that were controlled by the FGF21-AMPKα signaling pathway and found that upregulated mRNA levels of catalase (CAT), nuclear factor (erythroid-derived 2)-like 2 (Nrf-2) and heme oxygenase 1 (HO-1) by rFGF21 were greatly inhibited by both FIIN-4 and Compound C (Fig. 5f, S4F).

**Fig. 6:**
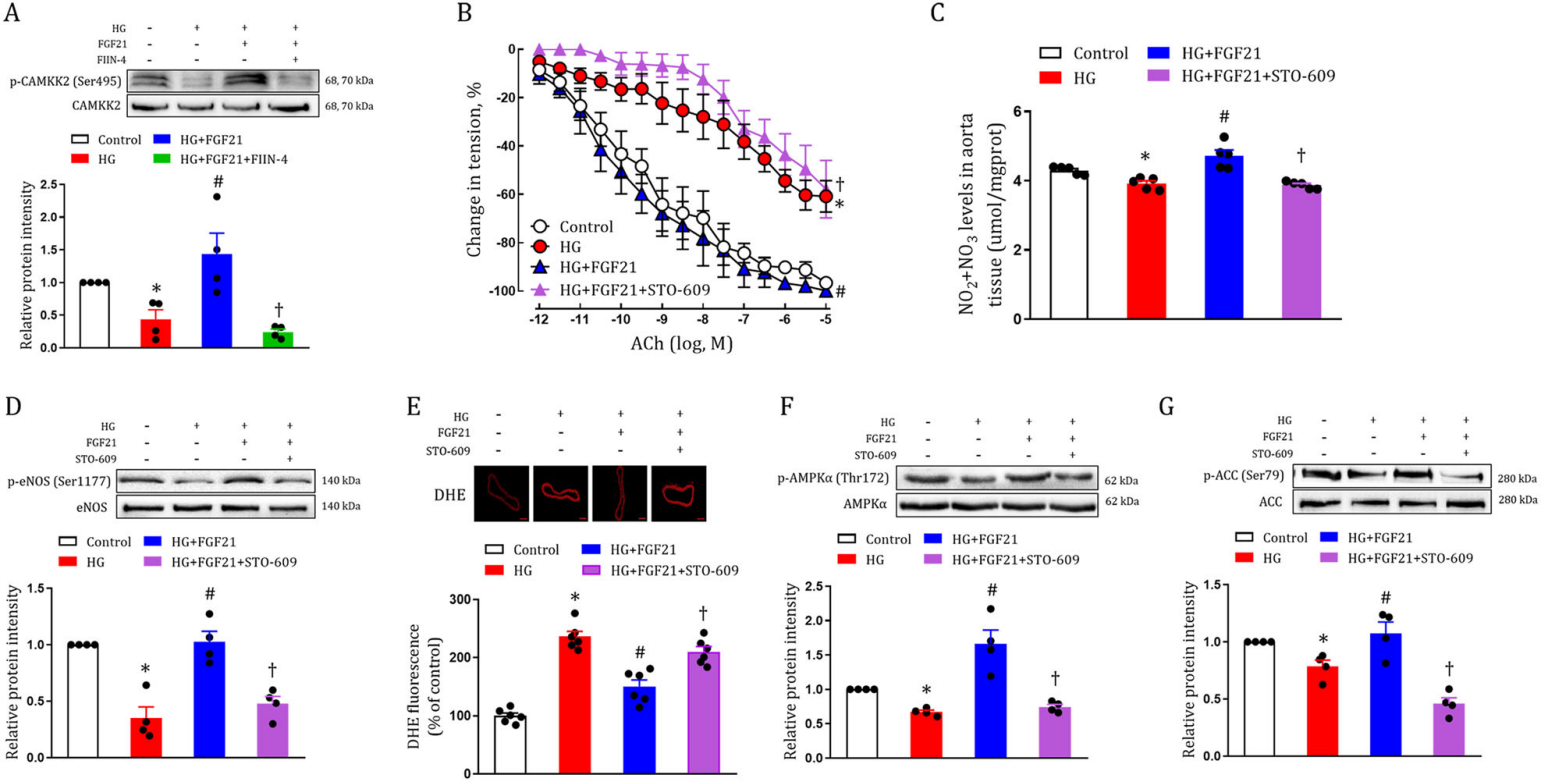
RFGF21 activates CaMKK2 to upregulate AMPKα activity, improve endothelium-dependent relaxation and alleviate oxidative stress in aortas challenged with HG. **a** Aortas isolated form C57BL/6J mice were pretreated with FIIN-4 (10 μM) for 30 mins and exposed to either HG (30 mM) alone or HG plus rFGF21 (0.01 mg/ml) for an additional 2 h. Phosphorylation level of CaMKK2 as determined by western blot analysis (upper panel) and quantitation using ImageJ software (lower panel) (*n* = 4). **b–g** Aortas isolated form C57BL/6J mice were pretreated with STO-609 (5 μg/ml) for 30 mins and exposed to either HG (30 mM) alone or HG plus rFGF21 (0.01 mg/ml) for an additional 2 h. **b** Dose-dependent relaxation to ACh (*n* = 5). **c** NO_2_ and NO_3_ levels stimulated by ACh (6 × 10^−8^ M) for 3 mins (*n* = 5). **d** Phosphorylation level of eNOS stimulated by ACh (6 × 10^−8^ M) for 3 mins as determined by western blot analysis (upper panel) and quantitation using ImageJ software (lower panel) (*n* = 4). **e** Immunofluorescent DHE staining (*n* = 6). The upper panel shows DHE staining and the lower panel shows quantitation using ImageJ software. Scale bars, 100 μm. **(f, g)** Phosphorylation level of AMPKα (**f**) and ACC (**g**) as determined by western blot analysis (upper panel) and quantitation using ImageJ software (lower panel) (*n* = 4). All data are presented as mean ± SEM. *p < 0.05 vs Control; ^#^*p* < 0.05 vs HG; ^†^*p* < 0.05 vs HG + FGF21

FGF21 triggers intracellular calcium release^31–33^, possibly leading to activation of AMPK by calcium/calmodulin-dependent protein kinase kinase 2 (CaMKK2, also known as CaMKKβ) pathway^34–36^. Indeed, we found that impaired activity of CaMKK2 by HG was reversed by co-administration with rFGF21 in isolated aortas, and this improvement was blocked by FIIN-4 (Fig. 6a). The alleviating effects of FGF21 on oxidative stress, endothelial dysfunction and reduced activations of AMPKα and ACC were substantially abrogated by the selective CaMKK2 antagonist (STO-609)^36,37^ in aorta or HUVEC (Figs. 6b–g, S6A–C). Taken together, the data suggest that FGF21 may directly bind FGFRs to activate the CaMKK2/AMPKα singling pathway to alleviate oxidative stress-induced endothelium dysfunction.

### rFGF21 directly induces relaxation of mice aorta via CaMKK2/AMPKα activation

**Fig. 7:**
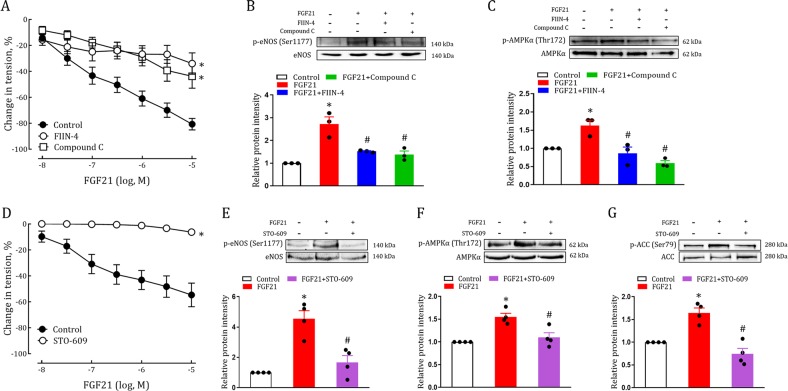
RFGF21 induces relaxation of aorta via CaMKK2/ AMPKα mediated activation of eNOS. **a** Dose-dependent relaxation of aorta from C57BL/6J mice to rFGF21 pretreated with FIIN-4 (10 μM) or Compound C (10 μM) for 30 mins (*n* = 7–12). **b**, **c** Phosphorylation level of eNOS (**b**) and AMPKα (**c**) in aortas from C57BL/6 J mice pretreated with FIIN-4 (10 μM) or Compound C (10 μM) for 30 mins and exposed to rFGF21 (0.01 mg/ml) for 3 mins as determined by western blot analysis (upper panel) and quantitation using ImageJ software (lower panel) (*n* = 3). **d** Dose-dependent relaxation of aorta from C57BL/6J mice to rFGF21 pretreated with STO-609 (5 μg/ml) for 30 mins (*n* = 9–10). **e–g** Phosphorylation level of eNOS (**e**), AMPKα (**f**) and ACC (**g**) in aortas from C57BL/6J mice pretreated with STO-609 (5 μg/ml) for 30 mins and exposed to rFGF21 (0.01 mg/ml) for 3 mins as determined by western blot analysis (upper panel) and quantitation using ImageJ software (lower panel) (*n* = 4). All data are presented as mean ± SEM. *p < 0.05 vs Control; ^#^*p* < 0.05 vs FGF21

As a cognate ligand for FGFR1, FGF2 induces endothelium-dependent dilation of coronary arterioles^38^. Activation of AMPKα also potently relaxes aorta by an endothelium-dependent mechanism^29,39^. Therefore, we hypothesized that FGF21 induced endothelium-dependent relaxation of mouse aorta through AMPKα activation in addition to its anti-oxidative capacity. Aorta was isolated from WT mice and treated with an increasing concentration of rFGF21. We found that rFGF21 dose-dependently induced marked relaxation in the aorta (Fig. 7a, d). Furthermore, this FGF21-mediated relaxation was mostly blocked by FIIN-4, Compound C and STO-609, suggesting that the FGF21-CaMKK2-AMPKα signaling pathway directly induces dilation in conduit arteries (Fig. 7a, d). This notion was further confirmed by the fact that the rFGF21-upregulated phosphorylation level of eNOS, AMPKα or ACC was largely abrogated by FIIN-4 (Fig. 7b, c), Compound C (Fig. 7b, c), AMPKα siRNA (Fig. S5E–H), or STO-609 (Figs. 7e–g, S6D–F) in isolated aorta or cultured HUVEC.

## Discussion

The restoration of vascular endothelial function is a pathway for amelioration of cardiovascular diseases that are main causes of mortality in diabetic patients. FGF21 is a hormone in the FGF superfamily that exerts several beneficial effects on metabolic diseases and their associated complications. However, the effects of FGF21 on impaired endothelial dysfunction of blood vessels in metabolic diseases remains unclear. This study shows that FGF21 improves dilation of aorta in both T1D and T2D mice, probably via CaMKK2/AMPKα-mediated suppression of oxidative stress and activation of eNOS.

The etiology and pathogenesis of T1D and T2D are quite different. The former refers to a state of absolute insulin deficiency, whereas the latter is a condition of insulin resistance^1,40,41^. In the present study, we found that FGF21 improves hyperglycemia, insulin resistance and endothelium-dependent relaxation of aorta in two T2D mouse models (HFD-STZ-induced and *db/db*). Although FGF21 failed to rescue high blood glucose in T1D, it remained effective in the alleviation of endothelial dysfunction. These results indicate that FGF21 may initiate a mechanism that is independent of its glucose-lowering and insulin-sensitizing effects. Indeed, we observed that FGF21 prevented HG-induced impairment of endothelial function in an in vitro model, suggesting that FGF21 directly acts on endothelial cells to exert its protective effect. This is further supported by the fact that both the receptor (FGFR1) and co-receptor (β-klotho) of FGF21 are expressed in vascular endothelial cells^25–27^. We also observed that certain levels of FGFR1 (with the highest bind affinity to FGF21) and β-klotho could be detected in the aortas of WT mice. Moreover, this notion is reinforced by the use of FGFR antagonist that blocked most of the biological effects of FGF21 in vitro.

Hyperglycemia is a common pathological change occurring both in T1D and T2D^3,22^. Several lines of evidence have shown that high glucose-induced oxidative stress in diabetes is a major etiologic factor of several diabetic complications including macrovasculopathy^3,22,42,43^. We found that oxidative stress was exacerbated in the endothelium of the aorta by hyperglycemia in diabetic models or by high glucose in vitro. Conversely, the worsened oxidative burden was mostly abrogated by FGF21. Furthermore, the inhibition of FGF21 on oxidative stress was abolished by FGFR antagonist. Given that FGF21 acts on endothelial cells, it is logical to postulate that the improvement of endothelial function by FGF21 is partially mediated by its direct inhibition on oxidative stress.

AMPK is a well-studied cellular energy sensor that is activated by various types of metabolic stresses to regulate energy metabolism within the cell and the body^30,44^. However, the activity of AMPK is dysregulated when exposed to persistent metabolic burdens such as is the case in metabolic syndrome or T2D^44^. By contrast, the activation of AMPK (physiological or pharmacological) improves insulin sensitivity and metabolic health^30,44^. In vascular endothelial cells, AMPK is activated to suppress oxidative stress-induced injury via various mechanisms^23,24^. Consistently, we also found that AMPK activity in aorta was suppressed in diabetic mice or challenged with high glucose that was in parallel with increased ROS production and impaired endothelial function. The administration of FGF21 increased phosphorylation levels of AMPKα, reduced oxidative stress and enhance vessel relaxation, all of which were mostly blocked by its antagonist or siRNA. We further explored the underlying anti-oxidative molecules that might be regulated by AMPKα and found that CAT, Nrf-2, and HO-1 (pivotal anti-oxidative stress enzymes) were potential candidates^45–47^. The data suggest that FGF21 may activate AMPKα to suppress oxidative stress and alleviate endothelial dysfunction.

In mammals, two major upstream kinases of AMPK are tumor suppressor LKB1 and CaMKK (especially CaMKK2)^30,36^. CaMKK2 induces activation of AMPK in response to increased intracellular calcium levels, which does not require any change in ADP or AMP levels^30,34–36^. FGF21 triggers intracellular calcium release^31–33^, thereby making it a possible upstream activator of CaMKK2. In the present study, we found that rFGF21 upregulated the activity of CaMKK2 by binding FGFR. By contrast, blockage of CaMKK2 by its selective antagonist substantially abrogated most of the beneficial effects of FGF21 on activating AMPKα, reducing oxidative stress and enhancing vessel relaxation. Taken together, the data suggest that CaMKK2 may be an upstream regulator determining the activity of AMPKα and mediating the alleviating effects of FGF21 on diabetes-induced oxidative stress and endothelial dysfunction.

Apart from its role in the inhibition of oxidative stress, AMPK activates endothelium-dependent vasorelaxation^29,39,48^. As a cognate ligand for FGFR1^49^, FGF2 is also able to elicit a vasodilation depending on endothelium activation^38^. In the present study, FGF21 activated CaMKK2, AMPKα and eNOS and induced dose-dependent dilation of aorta in control buffer. Furthermore, this vasorelaxation was abrogated by FGFR, CaMKK2 and AMPK antagonists, and AMPKα siRNA. Therefore, FGF21 induces relaxation of aorta through CaMKK2/AMPKα activation may also contribute to the alleviation of endothelial dysfunction in addition to its potent inhibition on oxidative stress.

**Fig. 8:**
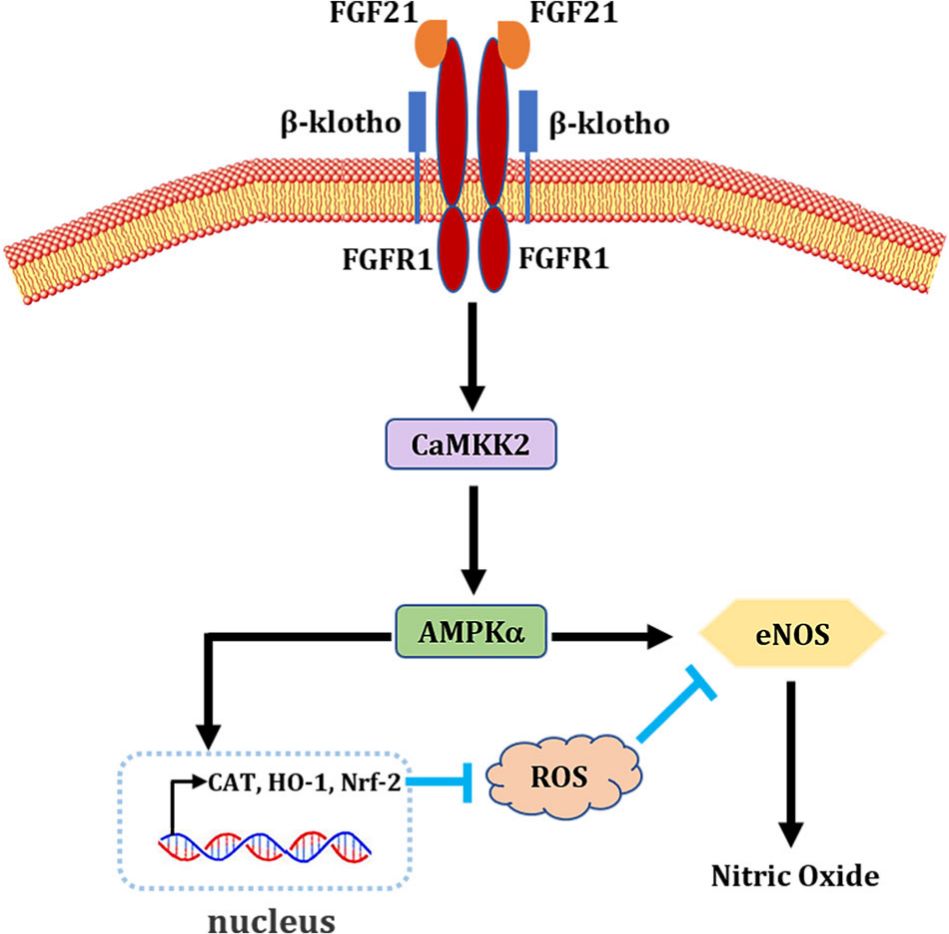
Schematic diagram of FGF21-mediated enhancement of endothelium-dependent relaxation. The normal function of vascular endothelial cell is impaired by oxidative stress that is initiated by metabolic disorders. FGF21 binds to FGFRs (i.e., FGFR1) to potentiate CaMKK2 and AMPKα activities. On the one hand, AMPKα upregulates the expressions of CAT, Nrf-2 and HO-1 to abrogate oxidative stress. On the other hand, AMPKα directly enhances the activity of eNOS. The strengthened eNOS activity leads to an increased production of nitric oxide and restoration of endothelium-dependent vasodilatation

Previous studies have shown an indirect regulation of FGF21 on vasculopathies, including atherosclerosis and hypertension, by acting on adipocytes, hepatocytes and renal cells^17,18^. In the present study, we found that FGF21 directly bound FGFR1 to activate CaMKK2 and AMPKα, to inhibit oxidative stress and to enhance eNOS phosphorylation levels in endothelial cells of large vessels. Furthermore, we found that CAT, Nrf-2, and HO-1 may be downstream targets activated by AMPK to suppress the oxidative burden exposed to HG. Taken together, our data provide a novel mechanistic understanding of the biological functions of FGF21 on vascular diseases challenged with metabolic disorders and demonstrate that FGF21 is a potent and effective protein to treat endothelial dysfunction through the direct suppression of oxidative stress and enhancement of eNOS signaling via CaMKK2/AMPKα activation in diabetes (Fig. 8).

## Materials and methods

### Expression and purification of rFGF21

Recombinant human FGF21 (rFGF21) (His29-Ser209) was expressed using the p-SUMO-FGF21 plasmid in *E. coli* and purified according to our previously published literature^50^.

### Animal welfare and animal model

Male *db/db* (C57BLKS/J-lepr^db^/lepr^db^) mice and their non-diabetic *db/m* littermates and male C57BL/6J mice were purchased from the Model Animal Research Center of Nanjing University (Nanjing, China). All protocols used in this study were approved by the Animal Care and Use Committee of Wenzhou Medical University, China. The animals were housed in an animal facility with controlled environment (22 ± 2 °C, 50–60% humidity, 12-h light/dark cycle, lights on at 7 a.m.) and free access to food and water. All animals were acclimatized to our laboratory environment before use. After all treatments, mice were anesthetized with diethyl ether and sacrificed by cervical dislocation.

HFD–STZ-induced T2D mice were established by feeding 8-week-old male C57BL/6 J mice with a high-fat diet (60 % fat; Research Diets, Inc, New Brunswick, NJ) for eight weeks to initiate insulin resistance. After that, the animals were introperitoneal (i.p.) injected with STZ (35 mg/Kg body weight) in citrate buffer (pH4.5) for three consecutive days; control mice received the same volume of citrate buffer. T1D was induced in 8-week-old male C57BL/6 J mice by i.p. injection of 100 mg/kg STZ for two days followed by three consecutive days injection of 60 mg/kg STZ. After two weeks, fed blood glucose levels of mice were determined using a FreeStyle complete blood glucose monitor (Abbott Diabetes Care Inc., Alameda, CA) and those with blood glucose ≥300 mg/dl (16.7 mM) were considered as diabetic mice.

### Functional evaluation of rFGF21 in diabetic mice

Before each study, mice were randomly assigned based on body weight and blood glucose level as determined above. All drugs and buffer controls were delivered by i.p. injection into mice. HFD-STZ-induced T2D mice and *db/db* mice were injected with rFGF21 for consecutive 33 days (0.5 mg/Kg body weight). T1D mice were injected with rFGF21 for consecutive 30 days (0.5 mg/Kg body weight). Blood samples were taken by tail snip measured every three days and glucose levels were determined as described above. Following the final dose, intraperitoneal glucose tolerance tests (IPGTTs) were conducted on HFD-STZ-induced T2D mice and *db/db* mice after fasting overnight (12 h); these mice were i.p. injected with a dextrose solution (1.0 g/kg body weight) and blood samples were taken at indicated time points and glucose levels were determined. Area under the curve (AUC) for IPGTTs was calculated by the trapezoid rule for the glucose tolerance curve using GraphPad Prism 8 software (GraphPad Software, San Diego, California). Serum levels of LDL, cholesterol, triglyceride, FFA, and HDL were measured according to protocols provided (Nanjing Jiancheng Bioengineering Institute, Nanjing, China).

### In vitro incubation study

Aorta rings (length: 3–5 mm) isolated from C57BL/6J mice were suspended in organ chambers filled with 10 ml or in tubes with 5 ml of modified Krebs-Ringer bicarbonate solution (composition (mM): NaCl, 118.3; KCl, 4.7; CaCl_2_, 2.5; MgSO_4_, 1.2; KH_2_PO_4_, 1.2; NaHCO_3_, 25.0; glucose, 11.1) maintained at 37 + /-1 °C and aerated with 95% O_2_ and 5% CO_2_ (pH = 7.4). After 30 mins equilibration, the rings were pretreated with FIIN-4 (10 μM), Compound C (10 μM), STO-609 (5 μg/ml) or buffer control for 30 mins (for both high glucose (HG) and rFGF21 incubations) and exposed to either HG (30 mM) alone or HG plus rFGF21 (0.01 mg/ml) for additional 2 h (for HG incubation).

For vessel tension studies, the vessels were preconstricted with norepinephrine (NE) (3 × 10^−7^ M) and dose-dependent relaxation responses of acetylcholine (ACh) or FGF21 were recorded. For intracellular signaling assays or measuring nitric oxide (NO) synthesis, the vessels were incubated with ACh (6 × 10^−8^ M) (for HG incubation) or FGF21 (0.01 mg/ml) (for rFGF21 incubation) for 3 mins.

### Vessel tension studies

Aorta rings (length: 3–5 mm) isolated from control, diabetic and rFGF21-treated mice were suspended in organ chambers filled with 10 ml of modified Krebs-Ringer bicarbonate solution maintained at 37 ± 1 °C and aerated with 95% O_2_ and 5% CO_2_ (pH = 7.4). Each ring was suspended through two stirrups passing through the lumen; one stirrup was anchored to the bottom of the organ chamber while the other connected to a strain gauge (RM6240 Systems/4sp, Chengdu Instrument Company, Chengdu, China) for measuring isometric force.

At the beginning, aorta ring was stretched to its optimal resting tension (~1.0 g) by step-wise stretching followed by an hour equilibration. Dose-dependent relaxation responses of ACh (10^−12^–10^−5^ M) or rFGF21 (10^−8^–10^−5^ M) was performed in rings preconstricted with NE (3 × 10^−7^ M) to a similar tension level.

### Cell culture and treatment

The human umbilical vascular endothelial cells (HUVECs) were purchased from Lonza and were cultured in endothelial cell growth medium (EGM^TM^-2 Endothelial Cell Growth Medium-2 BulletKit^TM^, cat. no. CC-3156 and CC-4176, Lonza, Basel, Switzerland). Fifth to seventh passage of subconfluent cells were used in the subsequent experiments.

For intracellular signaling assays, HUVECs were starved with serum-free low-glucose DMEM (cat. no. C11885500BT, Gibco, Thermo Fisher Scientific, Waltham, MA) for 12 h. Then, the cells were pretreated with STO-609 (5 μg/ml) or buffer control for 1 h (for both high glucose (HG) and rFGF21 incubations) and were exposed to either HG (35 mM) alone or HG plus rFGF21 (0.01 mg/ml) for an additional 6 h (for HG incubation). Subsequently, the cells were incubated with ACh (6 × 10^−8^ M) (for HG incubation) or FGF21 (0.01 mg/ml) (for rFGF21 incubation) for 10 mins and were lysed for subsequent measurement of levels of various downstream signals using western blotting.

For knockdown of AMPKα expression by siRNA, the cells were seeded in six-well plates and grown to 70% confluence. Transient transfections were performed using Lipofectamine 3000 Transfection Reagent (cat. no. L3000-008, Thermo Fisher Scientific, Waltham, MA) according to the manufacturer’s protocol. After cells were transfected with control (cat. no. sc-36869, Santa Cruz Biotechnology, Dallas, Texas, USA) or AMPKα siRNA (cat. no. sc-45312, Santa Cruz Biotechnology, Dallas, Texas, USA) for 36 h, they were starved for 12 h and treated as described above.

### Western blot analysis

Mice aortas, epididymal white adipose tissues (WAT(e)s) and HUVECs were homogenized in RIPA lysis buffer (25 mM Tris, pH 7.6, 150 mM NaCl, 1%NP-40, 1% sodium deoxycholate, 0.1% SDS) supplemented with protease and phosphatase inhibitors (all from Thermo Fisher Scientific Waltham, MA). Protein concentrations were determined using a BCA Kit (Protein Assay Kit, Beyotime Biotechnology, Shanghai, China). For determining phosphorylation and protein expression levels, equal quantities of soluble protein (50 μg) were separated using 10-12% SDS-PAGE and electro-transferred onto a nitrocellulose membrane. Protein blots were probed with primary antibodies against p-eNOS (anti-rabbit; dilution: 1:1000; cat. no. MA5-14957; Thermo Fisher Scientific, Waltham, MA), eNOS (anti-mouse; dilution: 1:3000; cat. no. ab50010; Abcam, Cambridge, MA), p-AMPKα (anti-rabbit; dilution: 1:1000; cat. no. 2535; Cell Signaling Technology, Danvers, MA), AMPKα (anti-rabbit; dilution: 1:1000; cat. no. 5831; Cell Signaling Technology, Danvers, MA), p-ACC (anti-rabbit; dilution: 1:1000; cat. no. 11818; Cell Signaling Technology, Danvers, MA), ACC (anti-rabbit; dilution: 1:1000; cat. no. 3662; Cell Signaling Technology, Danvers, MA), p-CaMKK2 (anti-rabbit; dilution: 1:1000; cat. no. 16737; Cell Signaling Technology, Danvers, MA), CaMKK2 (anti-rabbit; dilution: 1:1000; cat. no. 16810; Cell Signaling Technology, Danvers, MA), FGFR1 (anti-rabbit; dilution: 1:1000; cat. no. 9740; Cell Signaling Technology, Danvers, MA), β-klotho (anti-mouse; dilution: 1:500; cat. no. AF2619; Bio-Techne MN, USA) and GAPDH (anti-rabbit; dilution: 1:1000; cat. no. 2118; Cell Signaling Technology, Danvers, MA). Immune-reactive bands were detected by incubating with goat anti-mouse (dilution: 1:3000; cat. no. sc-2005; Santa Cruz Biotechnology, Dallas, TX) or anti-rabbit secondary antibody (dilution: 1:3000; cat. no. sc-2004; Santa Cruz Biotechnology, Dallas, TX) conjugated with horseradish peroxidase and visualized using enhanced chemiluminescence (ECL) reagents (Bio-Rad, Hercules, CA). After that, optical densities of the immunoblots were analyzed using ImageJ image software (version 1.38e, NIH, Bethesda, MD) and normalized to the scanning signals of their respective controls.

### Measurement of NO synthesis

Generation of NO was determined by measuring stable NO metabolites (i.e. total nitrites) in aortas after incubations and stimulated by ACh (6 × 10^−8^ M) using a nitrite detection kit (Beyotime, Shanghai, China). Briefly, 50 μl of tissue homogenate was mixed with 50 μl of Griess reagent in a 96-well plate. Nitrite concentration was determined by spectrophotometry (540 nm) from a standard curve (0–100 μmol/l) derived from NaNO_2_ and protein concentrations were determined as described above.

### DHE staining

The frozen sections (5 μm) of aortas were incubated with dihydroethidium (DHE) (1.5 mmol/L) for 30 mins and visualized using a fluorescence microscope (TCS-SP8, Leica, Germany).

### RNA extraction, cDNA synthesis, and quantitative RT-PCR

Total RNA was extracted from mouse aortas with TRIzol reagent (Thermo Fisher Scientific, Waltham, MA) and purified using a RNeasy Mini Kit (Qiagen, Valencia, CA). A Two-step M-MLV Platinum SYBR Green qPCR Super Mix-UDG kit (Thermo Fisher Scientific, Waltham, MA) was used for reverse transcription and quantitative PCR. GAPDH was used as an endogenous control to normalize differences in the quantity of total RNA added to each reaction. Primers were synthesized by Invitrogen (Thermo Fisher Scientific, Waltham, MA) as follows: CAT, forward 5′-TGGCACACTTTGACAGAGAGC-3′ and reverse 5′-CCTTTGCCTTGGAGTATCTGG-3′; Nrf-2, forward 5′-TCTTGGAGTAAGTCGAGAAGTGT-3′ and reverse 5’-GTTGAAACTGAGCGAAAAAGGC -3′; HO-1, forward 5′-AAGCCGAGAATGCTGAGTTCA-3′ and reverse 5′-GCCGTGTAGATATGGTACAAGGA-3′; GAPDH, forward 5′-AATGTGTCCGTCGTGGATCT-3′ and reverse 5′-CATCGAAGGTGGAAGAGTGG-3′.

### Statistical analyses

All results are expressed as mean ± SEM. Student’s unpaired *t* tests were used for the comparison of two groups. While student’s paired *t* tests were applied in the comparison of the same group before and after a treatment. One-way analysis of variance (ANOVA) test with Student–Newman–Keuls test for post hoc testing of multiple comparisons were used in the comparison of mean values of more than two groups. *P* value (two tailed) <0.05 is considered as statistical significance. *N* represents the number of replicates in the corresponding experiment.

## Supplementary information

A clean version of supplymentary informarion
