# Supplementary material for: Fibroblast growth factor 21 Ameliorates diabetes-induced endothelial dysfunction in mouse aorta via activation of the CaMKK2/AMPKα signaling pathway
Source: Cell Death Dis. 2019 Sep 11;10(9):665. doi: 10.1038/s41419-019-1893-6 (PMC6739326; doi:10.1038/s41419-019-1893-6)
Supplement: Supplementary file 1 — A clean version of supplymentary informarion [file 41419_2019_1893_MOESM1_ESM.pdf]

SUPPLEMENTARY INFORMATION

Fibroblast Growth Factor 21 Ameliorates Diabetes-induced Endothelial Dysfunction in Mouse Aorta via the Activation of CaMKK2/AMPKα Signaling Pathway

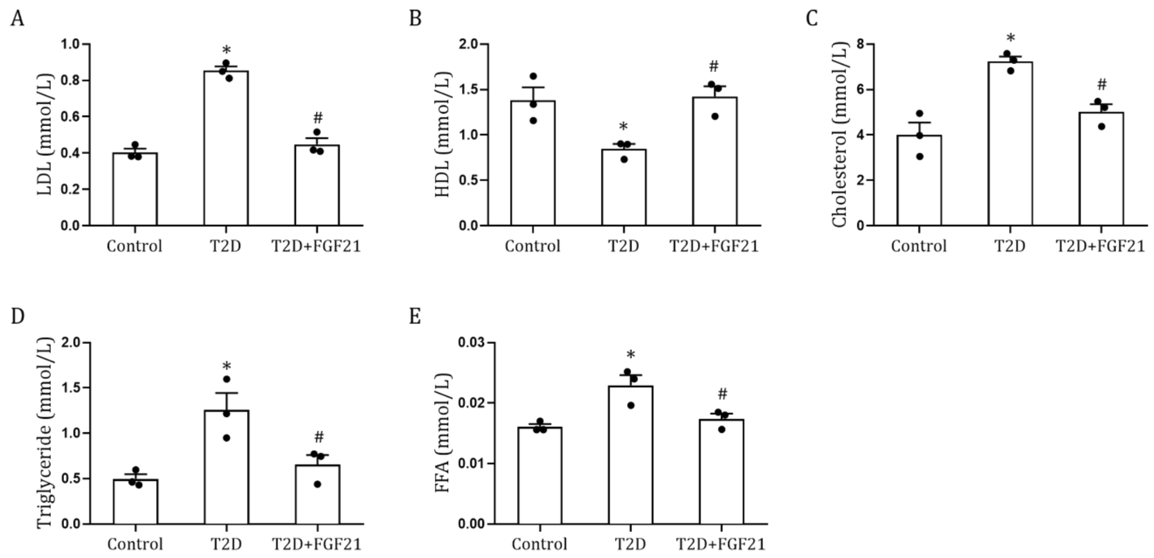

Figure S1. Long-term treatment of rFGF21 improves lipid metabolism in HFD-STZ Induced T2D mice

(A-E) LDL (A), cholesterol (C), triglyceride (D) and FFA (E) levels in serum were suppressed whereas HDL (B) level was upregulated in HFD-STZ induced T2D mice treated for 33 days with rFGF21 (0.5 mg/kg body weight) (n=3). All data are presented as mean +/- SEM. *$p<0.05$ vs Control; $^{\#}p<0.05$ vs T2D.

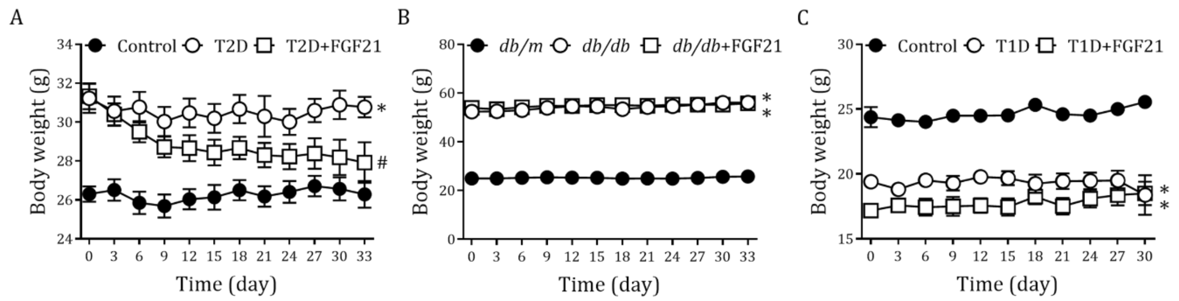

Figure S2. Effects of rFGF21 on body weights of HFD-STZ induced T2D, *db/db* and T1D mice

(A-C) Body weights of HFD-STZ induced T2D (A) (33 days) (n=9-10), *db/db* (B) (33 days) (n=5-6) or T1D mice (C) (30 days) (n=8-9) chronically treated with rFGF21 (0.5 mg/kg body weight). All data are presented as mean +/- SEM. *$p<0.05$ vs Control or *db/m*; [#]$p<0.05$ vs T2D.

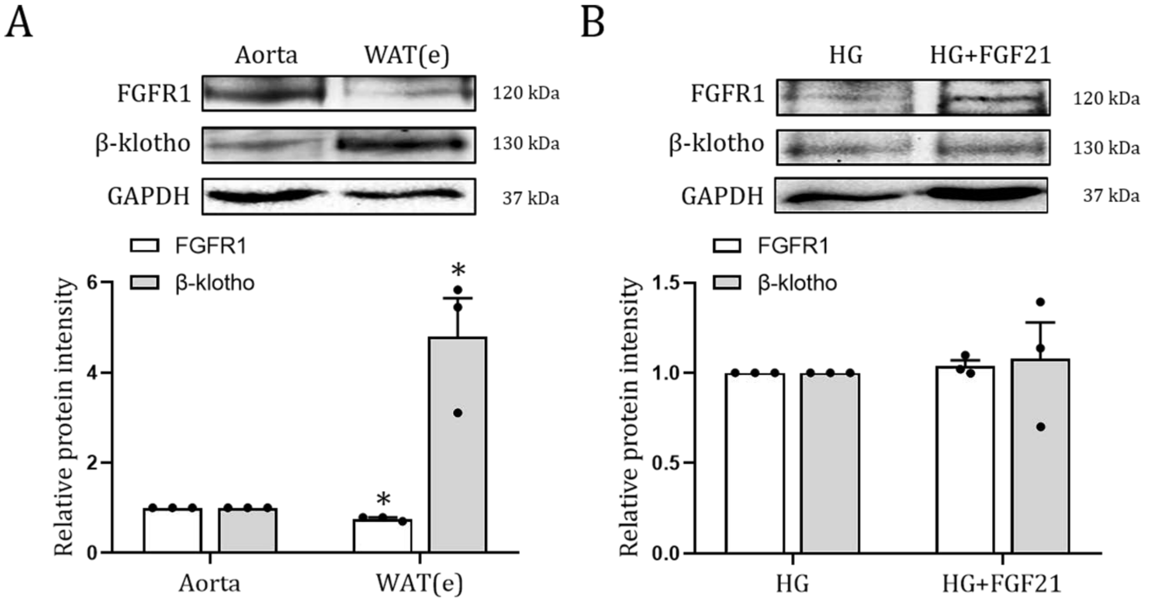

Figure S3. Protein expressions of FGFR1 and β-klotho in aortas and WAT(e) of C57BL/6J mice

(A) Protein expressions of FGFR1 and β-klotho in aortas and WAT(e) from C57BL/6J mice as determined by western blot analysis (upper panel) and quantitation using ImageJ software (lower panel) (n=3). All data are presented as mean +/- SEM. *p<0.05 vs Aorta.

(B) Protein expressions of FGFR1 and β-klotho in aortas of C57BL/6J mice treated with HG (30 mM) alone or HG plus rFGF21 (0.01 mg/ml) for 2 h as determined by western blot analysis (upper panel) and quantitation using ImageJ software (lower panel) (n=3). All data are presented as mean +/- SEM.

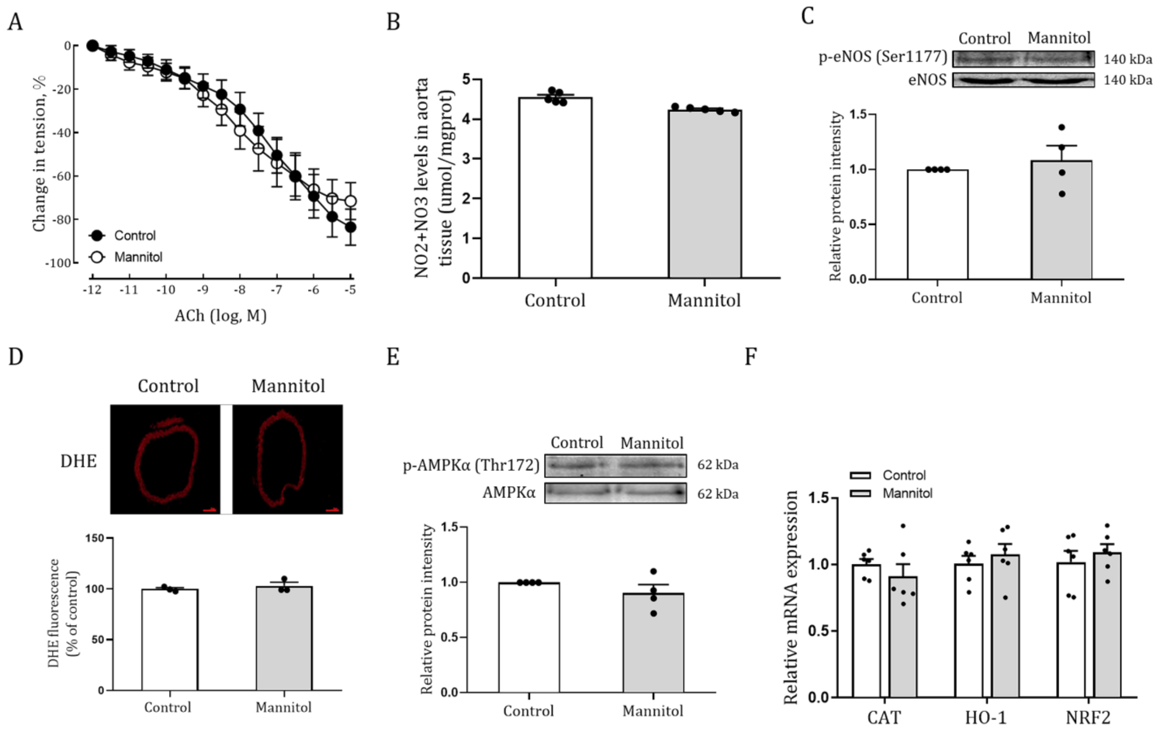

Figure S4. Mannitol does not affect endothelium dependent relaxation, ROS levels and AMPK signaling in aorta

(A-F) Aortas isolated form C57BL/6J mice were exposed to mannitol for 2 h. Kerb's buffer was served as control buffer. **(A)** Dose-dependent relaxation to ACh (n=6). **(B)** NO_2 and NO_3 levels stimulated by ACh ($6*10^{-8}$ M) for 3 mins (n=5). **(C)** Phosphorylation level of eNOS stimulated by ACh ($6*10^{-8}$ M) for 3 mins as determined by western blot analysis (upper panel) and quantitation using ImageJ software (lower panel) (n=4). **(D)** Immunofluorescent DHE staining (n=3). The upper panel shows DHE staining and the lower panel shows quantitation using ImageJ software. Scale bars, 100 μm. **(E)** Phosphorylation level of AMPKα as determined by western blot analysis (upper panel) and quantitation using ImageJ software (lower panel) (n=4). **(F)** mRNA levels of CAT, HO-1 and Nrf-2 as determined by RT-qPCR (n=6). All data are presented as mean +/- SEM.

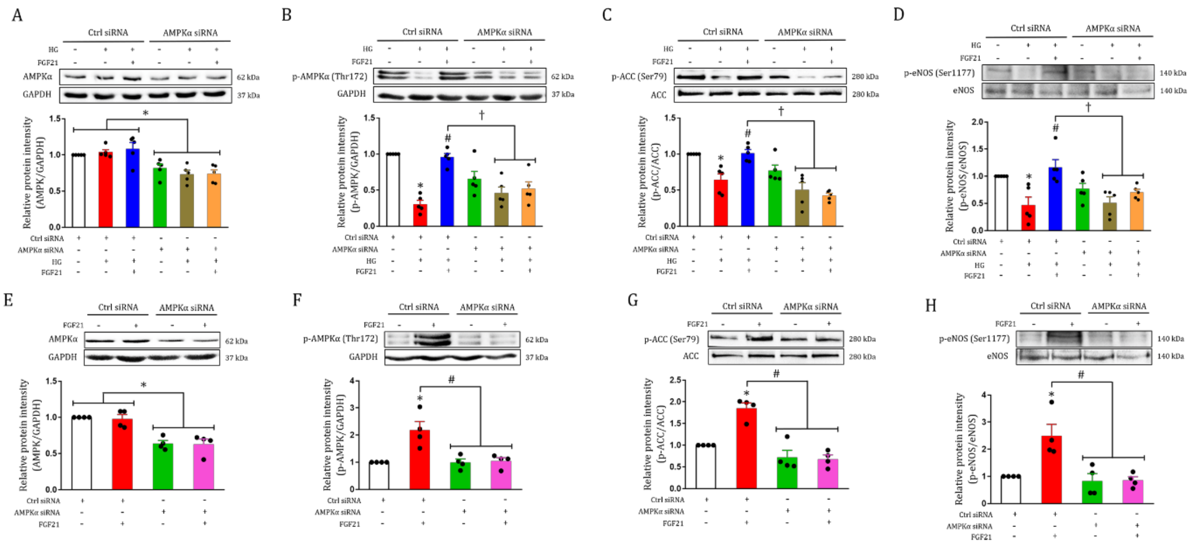

Figure S5. Knockdown of AMPKα abrogates activations of AMPKα, ACC and eNOS by rFGF21

(A-D) Human umbilical vascular endothelial cells (HUVECs) were transfected with control or AMPKα siRNA and exposed to either HG (35 mM) alone or HG plus rFGF21 (0.01 mg/ml) for an additional 6 hrs. **(A-C)** Protein expression (A) and phosphorylation level (B) of AMPKα and phosphorylation level of ACC (C) as determined by western blot analysis (upper panel) and quantitation using ImageJ software (lower panel) (n=5). **(D)** Phosphorylation level of eNOS stimulated by ACh ($6*10^{-8}$ M) for 10 mins as determined by western blot analysis (upper panel) and quantitation using ImageJ software (lower panel) (n=5). All data are presented as mean +/- SEM. *p<0.05 vs Ctrl siRNA, Ctrl siRNA+HG or Ctrl siRNA+HG+FGF21; #p<0.05 vs Ctrl siRNA+HG; †p<0.05 vs Ctrl siRNA+HG+FGF21. **(E-H)** HUVECs were transfected with control or AMPKα siRNA and exposed to exposed to rFGF21 (0.01 mg/ml) for 10 minutes. Protein expression (E) and phosphorylation level (F) of AMPKα and phosphorylation levels of ACC (G) and eNOS (H) as determined by western blot analysis (upper panel) and quantitation using ImageJ software (lower panel) (n=4).All data are presented as mean +/- SEM. *p<0.05 vs Ctrl siRNA or Ctrl siRNA+FGF21; #p<0.05 vs Ctrl siRNA+FGF21.

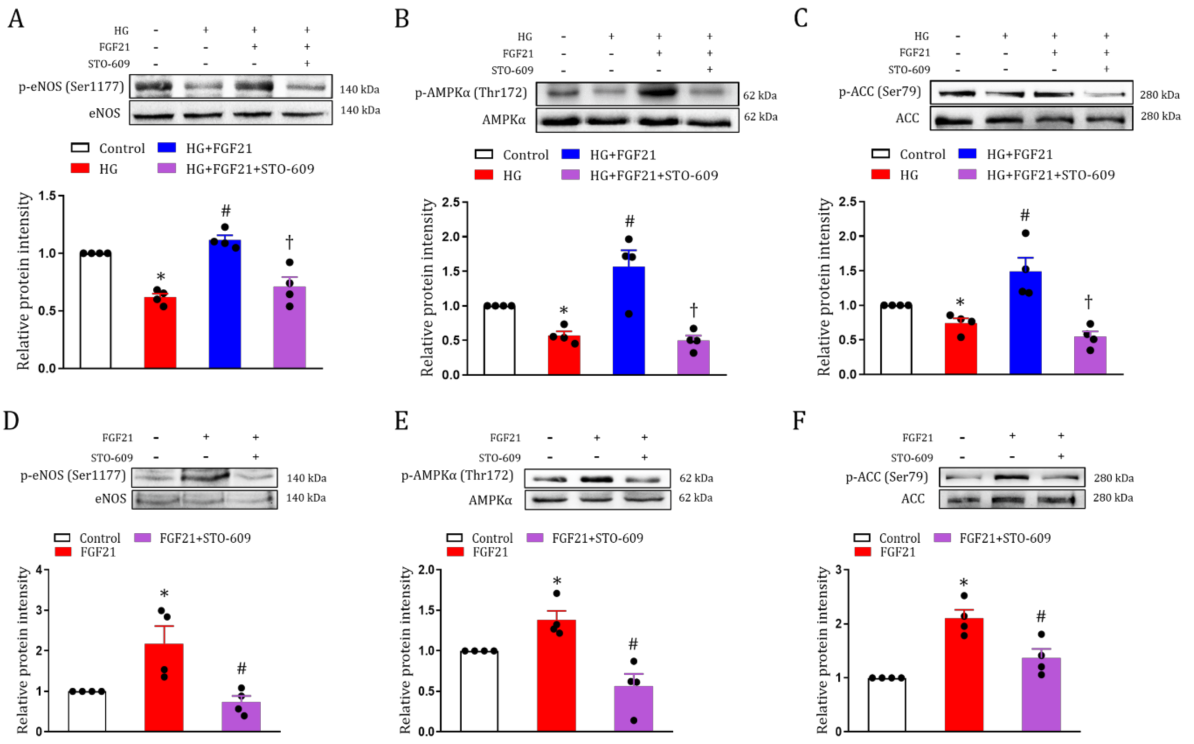

Figure S6. RFGF21 activates CaMKK2 to upregulate AMPKα and eNOS activities in HUVECs

(A-C) HUVECs were pretreated with STO-609 (5 µg/ml) for 1 h and exposed to either HG (35 mM) alone or HG plus rFGF21 (0.01 mg/ml) for an additional 6 hrs. **(A)** Phosphorylation level of eNOS stimulated by ACh ($6*10^{-8}$ M) for 10 mins as determined by western blot analysis (upper panel) and quantitation using ImageJ software (lower panel) (n=4). **(B, C)** Phosphorylation levels of AMPKα and ACC as determined by western blot analysis (upper panel) and quantitation using ImageJ software (lower panel) (n=4). All data are presented as mean +/- SEM. *$p<0.05$ vs Control; #$p<0.05$ vs HG; †$p<0.05$ vs HG+FGF21. **(D-F)** HUVECs were pretreated with STO-609 (5 µg/ml) for 1 h and exposed to rFGF21 (0.01 mg/ml) for 10 minutes. Phosphorylation levels of eNOS (D), AMPKα (E) and ACC (F) as determined by western blot analysis (upper panel) and quantitation using ImageJ software (lower panel) (n=4). All data are presented as mean +/- SEM. *$p<0.05$ vs Control; #$p<0.05$ vs FGF21.
